# Elevated mean neutrophil volume represents altered neutrophil composition and reflects damage after myocardial infarction

**DOI:** 10.1007/s00395-015-0513-6

**Published:** 2015-10-14

**Authors:** G. P. J. van Hout, W. W. van Solinge, C. M. Gijsberts, M. P. J. Teuben, P. H. C. Leliefeld, M. Heeres, F. Nijhoff, S. de Jong, L. Bosch, S. C. A. de Jager, A. Huisman, P. R. Stella, G. Pasterkamp, L. J. Koenderman, I. E. Hoefer

**Affiliations:** Laboratory of Experimental Cardiology (room G02.523), University Medical Centre Utrecht, Heidelberglaan 100, PO Box 85500, 3508 GA Utrecht, The Netherlands; Department of Clinical Chemistry and Hematology, University Medical Center Utrecht, Utrecht, The Netherlands; ICIN, Netherlands Heart Institute, Utrecht, The Netherlands; Department of Respiratory Medicine, University Medical Center Utrecht, Utrecht, The Netherlands; Department of Cardiology, University Medical Center Utrecht, Utrecht, The Netherlands

**Keywords:** Myocardial infarction, Ischemia–reperfusion injury, Neutrophil biology, Neutrophil subsets

## Abstract

Myocardial infarction (MI) induces an inflammatory response in which neutrophils fulfill a prominent role. Mean neutrophil volume (MNV) represents the average size of the circulating neutrophil population. Our goal was to determine the effect of MI on MNV and investigate the mechanisms behind MNV elevation. MNV of 84 MI patients was compared with the MNV of 209 stable angina patients and correlated to simultaneously measured CK levels. Fourteen pigs were subjected to temporary coronary balloon occlusion and blood was sampled at multiple time points to measure MNV. Echocardiography was performed followed by ex vivo infarct size assessment after 72 h. MNV was higher in MI patients compared to stable angina patients (602 SD26 AU vs. 580 SD20 AU, *p* < 0.0001) and correlated with simultaneously measured CK levels (*R* = 0.357, *p* < 0.0001). In pigs, MNV was elevated post-MI (451 SD11 AU vs. 469 SD12 AU), *p* < 0.0001). MNV correlated with infarct size (*R* = 0.705, *p* = 0.007) and inversely correlated with left ventricular ejection fraction (*R* = −0.718, *p* = 0.009). Cell sorting revealed an increased presence of banded neutrophils after MI, which have a higher MNV compared to mature neutrophils post-MI (495 SD14 AU vs. 478 SD11 AU, *p* = 0.012). MNV from coronary sinus blood was higher than MNV of neutrophils from simultaneously sampled arterial blood (463 SD7.6 AU vs. 461 SD8.6 AU, *p* = 0.013) post-MI. The current study shows MNV is elevated and reflects cardiac damage post-MI. MNV increases due to altered neutrophil composition and systemic neutrophil activation. MNV may be an interesting parameter for prognostic assessment in MI and provide new insights into pathological innate immune responses evoked by ischemia–reperfusion.

## Introduction

Ischemic damage after myocardial infarction (MI) induces a detrimental inflammatory response [1, 25, 32]. The best treatment to salvage myocardium post-MI is to restore myocardial reperfusion through percutaneous coronary intervention (PCI) [26]. Apart from myocardial salvage, reperfusion also allows for immediate interaction between the damaged myocardium and circulating cells, among which neutrophils are the first responders [13, 22, 48, 53].

After myocardial reperfusion, circulating cells migrate to the infarcted tissue to clear out necrotic cells and orchestrate cardiac wound healing [18, 48]. Paradoxically, the influx of inflammatory cells into the myocardium can result in the elimination of viable cardiomyocytes, thereby inducing infarct expansion [4, 36, 41]. Many clinical studies have shown that the severity of this inflammatory response is reflected by systemically measurable inflammatory parameters [27]. Especially circulating neutrophils have been shown to represent the amount of inflicted damage, since neutrophil numbers and ratios are associated with a worse prognosis and more adverse events after MI [11, 12, 19, 21, 31, 37–39, 47].

Currently, prognostic value of neutrophils to predict the outcome after MI is limited to neutrophil numbers or derivatives hereof, like neutrophil/lymphocyte ratio [2, 43]. Studies that focus on morphological changes of neutrophils, or use, e.g., membrane proteins as a prognostic marker are scarce. The reasons may be manifold. Amongst technical and cost issues, neutrophils have classically been regarded as a uniform population with detrimental effects in MI-related cardiac injury. However, new data show the existence of neutrophil subtypes, like banded and hypersegmented neutrophils, with distinct functional capacity that is reflected by morphological differences and receptor expression patterns [8, 42]. Morphological changes in the neutrophil population may therefore represent an altered circulating neutrophil composition during MI and investigating these characteristics will contribute to the understanding of phenotypic and functional differences of neutrophil subsets in MI.

Mean neutrophil volume (MNV) represents the average size of the circulating neutrophil population [3]. In contrast to many other morphological characteristics, MNV can be easily determined by clinically implemented automated hematological cell analyzers [45]. Moreover, changes in MNV have been proven to be associated with an increased inflammatory response and is a marker of disease severity in several infectious diseases and trauma, independent of neutrophil numbers [3, 9, 10, 28, 29, 34, 54].

Since MI induces an inflammatory response, and MNV is known to be elevated in inflammation-related diseases, in the current study we hypothesize that MNV changes after myocardial infarction in patients suffering from MI. We also hypothesize that possible MNV elevation reflects the extent of cardiac damage and is caused by an altered circulating neutrophil composition. To this extent, we investigated the effect of myocardial ischemia–reperfusion injury on MNV in a porcine model of MI and determined if MNV could be a possible marker in the setting of MI.

## Methods

### Patient sampling

To determine MNV after MI, blood samples from ST-elevation MI (STEMI) patients were compared with samples from stable angina patients and patients without coronary artery disease. Eighty-four STEMI patients included in the DEB-AMI trial (ClinicalTrials.gov identifier NCT00856765) were retrospectively selected. The exact inclusion and exclusion criteria of the DEB-AMI study cohort are described elsewhere [5]. Most importantly, patients were eligible for inclusion in the DEB-AMI trial only when having evidence of a single culprit lesion in the target vessel and received a PCI within 12 h after the onset of complaints. This minimizes the chances for confounding effects of previous ischemia on MNV. Patients with peri-procedural cardiac arrest were excluded. Patients between 18 and 80 years of age, suffering from STEMI (diagnosed by the presence of anginal complaints and >1 mm ST elevation in >2 contiguous leads or new left bundle branch block) between 2009 and 2013, from whom a white blood cell count was available within 72 h after the onset of complaints, were included.

As non-ischemic control groups, patients without coronary artery disease and stable angina patients with significant coronary artery disease were selected from the UCORBIO cohort (clinicaltrials.gov identifier: NCT02304744), a biobank of patients undergoing coronary angiography in the University Medical Center in Utrecht, the Netherlands. From 2011 to 2013, patients presenting without coronary artery disease (no CAD, *n* = 80) and patients with stable complaints (either stable angina, dyspnea complaints or silent ischemia) were enrolled from the catheterization laboratories (stable AP, *n* = 209) [14].

In all patients, MNV was measured by the Cell-Dyn Sapphire (CD-Sapphire), an automated hematological analyzer (see below). Creatine kinase (CK) was measured routinely after PCI in STEMI patients. Data for this study were obtained from the Utrecht Patient Oriented Database (UPOD). UPOD comprises information on patient demographics, hospital discharge diagnoses, medical procedures, medication orders and laboratory tests for all patients treated at the UMC Utrecht. The UPOD database is described in detail elsewhere [6]. All patients provided written informed consent. This study conforms to the declaration of Helsinki. Medical Review Board approval for this research project was obtained at the UMC Utrecht.

### Porcine sampling

All animal experiments were approved by the institutional animal welfare committee and were executed conforming to the ‘Guide for the Care and Use of Laboratory Animals’.

A total of 19 female landrace pigs were used in this study. Fourteen pigs (body weight 70.1 SD3.8 kg) were subjected to closed-chest left anterior descending artery (LAD) occlusion for 75 min followed by 3 days of reperfusion. Arterial blood was collected at baseline, at the end of the ischemic period and at multiple time points after reperfusion (0, 15, 30, 60 and 120 min) and was collected in ethylenediaminetetraacetic (EDTA) containing vacutainers followed by the measurements of MNV with the same type of hematology analyzer that was used for the analysis of human samples. From 4 pigs, additional blood samples were drawn at 4 and 8 h reperfusion. Furthermore, coronary sinus blood sampling was performed 15 min after reperfusion simultaneously with arterial blood sampling. After 72-h follow-up, pigs were re-anesthetized and 3D-echocardiography was performed after sternotomy. Five pigs were used for sham procedures, undergoing the same procedures except myocardial ischemia induction.

### Surgical procedure

Pre-treatment and anesthesia protocols have been described in detail elsewhere [20, 24]. In short, all animals were pre-treated with acetylsalicylic acid for 1 day (320 mg loading dose, 80 mg/day maintenance), clopidogrel for 3 days (75 mg/day) and amiodaron for 10 days (1200 mg loading dose, 800 mg/day maintenance). All medication was continued until the end of the 72-h follow-up period. Animals were anesthetized with an intramuscular injection of 0.4 mg/kg midazolam, 10 mg/kg ketamine and 0.014 mg/kg atropine. Venous access was obtained by insertion of an 18G cannula in the ear vein for intravenous administration of 5 mg/kg sodiumthiopental. Anesthesia was maintained with intravenous infusion of 0.5 mg/kg/h midazolam, 2.5 µg/kg/h sufentanyl and 0.1 mg/kg/h pancuronium. Pre-operatively, animals received a fentanyl patch (25 µg/h). Arterial access was obtained by introduction of an 8F sheath into the carotid artery after surgical exposure. A coronary angiogram of the left coronary tree was acquired using an 8F JL4 guiding catheter (Boston Scientific, Natick, MA, USA). An adequately sized balloon was placed distal to the second or third diagonal branch, depending on the anatomy of the coronary artery, and inflated for 75 min. Animals were observed for 3 h post-reperfusion and a permanent catheter was placed in the jugular vein in 4 pigs to allow venous blood sampling at 4 and 8 h reperfusion. The surgical wound was closed and animals were weaned from anesthesia. Animals were defibrillated in case of ventricular fibrillation (VF).

### Echocardiography, infarct size and troponin

Three-dimensional echocardiography was performed as described before [20, 24]. In short, pigs were re-anesthetized according to the same protocol after 72 h. Medial sternotomy was performed and a gel-filled flexible sleeve was placed directly on the apex of the heart. An X3-1 transducer on an iE33 ultrasound device (Philips, Eindhoven, The Netherlands) was used to perform the echocardiogram. Images were analyzed offline using QLab 10.1 (3DQ advanced) analysis software. Due to incomplete capture of the LV, one animal was excluded from the analysis. After 3D-echocardiography, animals were killed by exsanguination under anesthesia. The heart was excised and the LV was cut into 5 equal slices from apex to base. Slices were incubated in 1 % TTC (Sigma-Aldrich Chemicals, Zwijndrecht, the Netherlands) in 37 °C 0.9 %NaCl for 10 min to discriminate between infarct tissue and viable myocardium. After incubation, photographs of the slices were made and the infarct size as a ratio of the left ventricle (LV) was quantified using ImageJ software (NIH, Bethesda, MD, USA). Troponin was measured in plasma isolated from blood drawn after 2 h reperfusion, using a UniCel DxI Immunoassay system (Beckman Coulter, Brea, CA, USA) with a paramagnetic particle, chemiluminescent immunoassay (Beckman Coulter, Brea, CA, USA).

### Sham experiments

Five animals were subjected to sham experiments. Pre-treatment and anesthesia were similar to the protocol described above. Arterial access was again obtained by introduction of an 8F sheath into the carotid artery after surgical exposure. A coronary angiogram of the left coronary tree was acquired using an 8F JL4 guiding catheter (Boston scientific, Natick, MA, USA). Animals were observed for 6 h (corresponding to the observation period of the animals subjected to MI), followed by exsanguination.

### Cell-Dyn Sapphire—MNV measurements

For the measurements of MNV in both patients and pigs, the Cell-Dyn Sapphire (Abbott diagnostics, Santa Clara, CA, USA) was used. This device is a routine hematology analyzer, which allows for whole-blood analysis using spectrophotometry, electrical impedance and laser light scattering (multi-angle polarized scatter separation, MAPSS) to classify blood cells (platelets, erythrocytes and leukocytes). The device comprises detectors that are used for optical light scattering to measure different parameters that represent morphological characteristics (e.g., cellular volume, granularity, nuclear shape) of cells. Based on combinations of these parameters, cells are automatically classified into different subpopulations (neutrophils, monocytes, lymphocytes, eosinophils and basophils) according to pre-set gates incorporated into the software of the device [17, 35, 45]. For the measurements of MNV, a routine white blood cells count was performed for both patient and pig samples.

### Flow cytometry measurements in STEMI patients

To assess if STEMI patients showed increased circulating neutrophil subsets, flow cytometry analysis in two patients was performed. Blood was collected in EDTA-filled vacutainers and co-incubated with a mouse anti-human CD62L-ECD monoclonal antibody (Beckman Coulter, Brea, CA, USA) and a monoclonal CD16-APC-A700 antibody (Beckman Coulter, Brea, CA, USA) for 30 min. After incubation, blood was lysed for 10 min using Optilyse C (Beckman Coulter, Brea, CA, USA) followed by flow-cytometry measurements on the Gallios™ system (Beckman Coulter, Brea, CA, USA).

### Identification of neutrophil subsets in pigs

For the identification of neutrophil subsets in pigs, measurements of CD62L/CD16 expression on porcine neutrophils was performed using the Cell-Dyn Sapphire in samples drawn from a subset of 4 pigs. A supplementary analyzer processing option normally used for the automated determination of CD3^+^ CD4^+^ T-Helper and CD3^+^ CD8^+^ T-Suppressor cells, uses a modified analytical approach that combines simultaneous measurements of optical (necessary to determine MNV) and fluorescent (FL1/FL2, for CD62L/CD16 expression analysis) characteristics [15, 23]. Whole blood samples were co-incubated for 20 min with a commercially obtained directly labeled mouse anti-pig monoclonal antibodies against FcγRIII [CD16- phycoerythrin (PE), clone G7, AbdSerotec, Germany)] and, recently by our laboratory developed, mouse anti-pig monoclonal antibody against L-selectin (CD62L) (Abmart Inc. Shanghai, China). The unconjugated CD62L antibody was labeled with Alexa-488 (Life Technologies, Carlsbad, CA, USA) followed by automated measurements on the hematology analyzer.

### Porcine cells sorting and visualization

For morphological examination of neutrophil subsets, additional blood samples (*n* = 4) were collected in EDTA vacutainers. Red blood cells were lysed using 4 °C isotonic NH_4_Cl. Remaining white blood cells were washed with phosphate buffered saline supplemented with sodium citrate (0.4 % wt/vol) and pasteurized plasma protein solution (10 % vol/vol) (PBS2+). After washing and centrifugation at 1500 rpm for 5 min at 4 °C, cells were resuspended in PBS2+ and kept on ice until incubation with antibodies. Resuspended cells were incubated at 4 °C for 45 min with commercially obtained directly labeled mouse anti-pig monoclonal antibodies against FcyRIII (CD16-phycoerythrin (PE), clone G7, AbDSerotec, Germany) as well as the same mouse anti-pig monoclonal antibody against L-selectin (CD62L) as described before. Thereafter cells were washed and sorted on a MoFloAstrios (Beckman Coulter, Brea, CA, USA). Blood neutrophils were identified by their distinct forward and sideward scatter profiles. This yielded a neutrophil purity over 98 %. Thereafter, subsets were sorted based on their CD16 and CD62L expression patterns. In order to determine morphological characteristics of PMN subsets, a total of 100,000 cells per subset were isolated and used for cytospin slides and stained with May-Gruenwald & Giemsa.

### Determination of MNV after local activation of neutrophils

Porcine whole blood from healthy pigs (*n* = 4) was stimulated in vitro with either different concentrations of lipopolysaccharide (LPS, Sigma, *Escherichia coli* 055:B5) (10, 100 ng, 1 µg) or phosphate buffered saline (control). After 2 h of incubation, samples were automatically measured by the automated hematological analyzer to determine MNV as described before. To assess if the damaged myocardium could influence MNV levels, we performed coronary sinus sampling 15 min after reperfusion in pigs subjected to MI. A 4F-catheter was temporarily placed in the coronary sinus through an introducer sheath in the jugular vein to draw blood 15 min after reperfusion while blood was drawn simultaneously from the aorta as a reference.

### Data analysis and statistics

Files from human samples were analyzed by the internal Cell-Dyn Sapphire algorithm according to pre-set gates for different leukocyte subsets. Data from porcine samples were extracted and analyzed with manually set gates (Kaluza, Beckman Coulter, Brea, CA, USA), since the incorporated pre-set gates of the hematological analyzer were not suitable for porcine circulating cells. Data are expressed as means ± standard deviations unless mentioned otherwise. Groups were compared using a one-way ANOVA in case of more than two groups, a Student’s *t* test for unrelated measurements in case of comparisons of two separate groups and a paired Student’s *t* test for related measurements. Correlations were tested with Pearson’s correlation test.

## Results

### MNV is elevated in patients post-MI

**Fig. 1:**
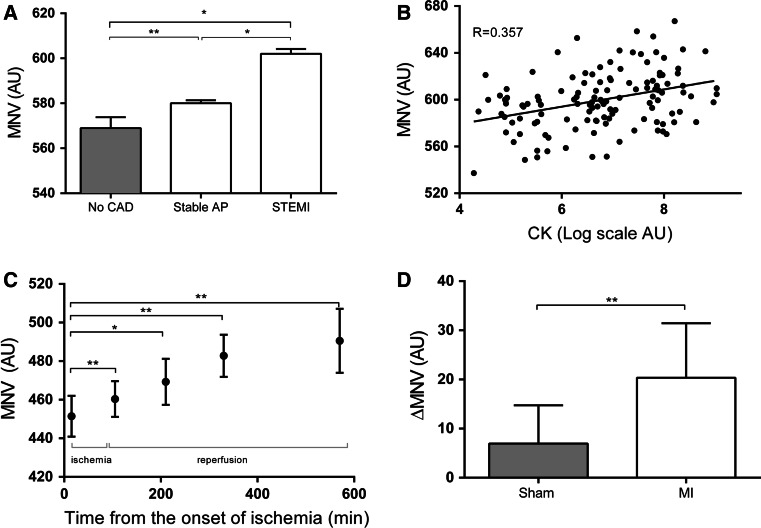
MNV is elevated after MI. **a** MNV is higher in STEMI patients compared to stable angina patients and patients without coronary artery disease. **b** MNV correlates with log-transformed CK levels in STEMI patients. **c** MNV is elevated in pigs after subjection to myocardial infarction. **d** MNV is higher in pigs subjected to MI than in pigs undergoing sham operation after a similar observation period. *CAD* coronary artery disease, *p < 0.0001 by one-way ANOVA, **p < 0.05

MNV was significantly higher in STEMI patients (*n* = 84) compared to stable angina patients (*n* = 209) and patients without coronary artery disease (*n* = 80) that served as control groups (STEMI: 602 SD26 arbitrary units (AU) vs. stable AP: 580 SD20 AU, no CAD: 568 SD43 AU, *p* < 0.0001) (Fig. 1a). MNV also correlated significantly with simultaneously measured CK levels (*R* = 0.357, *p* < 0.0001) in STEMI patients (Fig. 1b).

### MNV is elevated in pigs post-MI

To investigate the change of MNV in a controlled, prospective setting, 14 pigs were subjected to MI. One pig died due to resistant VF. In the remaining 13 pigs, MNV was elevated in a time-dependent manner post-MI (451 SD11 AU (baseline, *n* = 13) vs. 460 SD9 AU, *p* = 0.011 (15 min reperfusion, *n* = 13) vs. 469 SD12 AU, *p* < 0.0001 (2 h reperfusion, *n* = 13) vs. 483 SD11 AU, *p* = 0.009 (4 h reperfusion, *n* = 4) vs. 490 SD17 AU, *p* = 0.014 (8 h reperfusion, *n* = 4) (Fig. 1c). Moreover, ΔMNV between baseline and follow-up measurements differed significantly when comparing pigs subjected to MI and sham-operated pigs after a similar follow-up period of 4 h (20.4 SD11.1 AU vs. 7.0 SD7.8 AU, *p* = 0.026) (Fig. 1d).

**Fig. 2:**
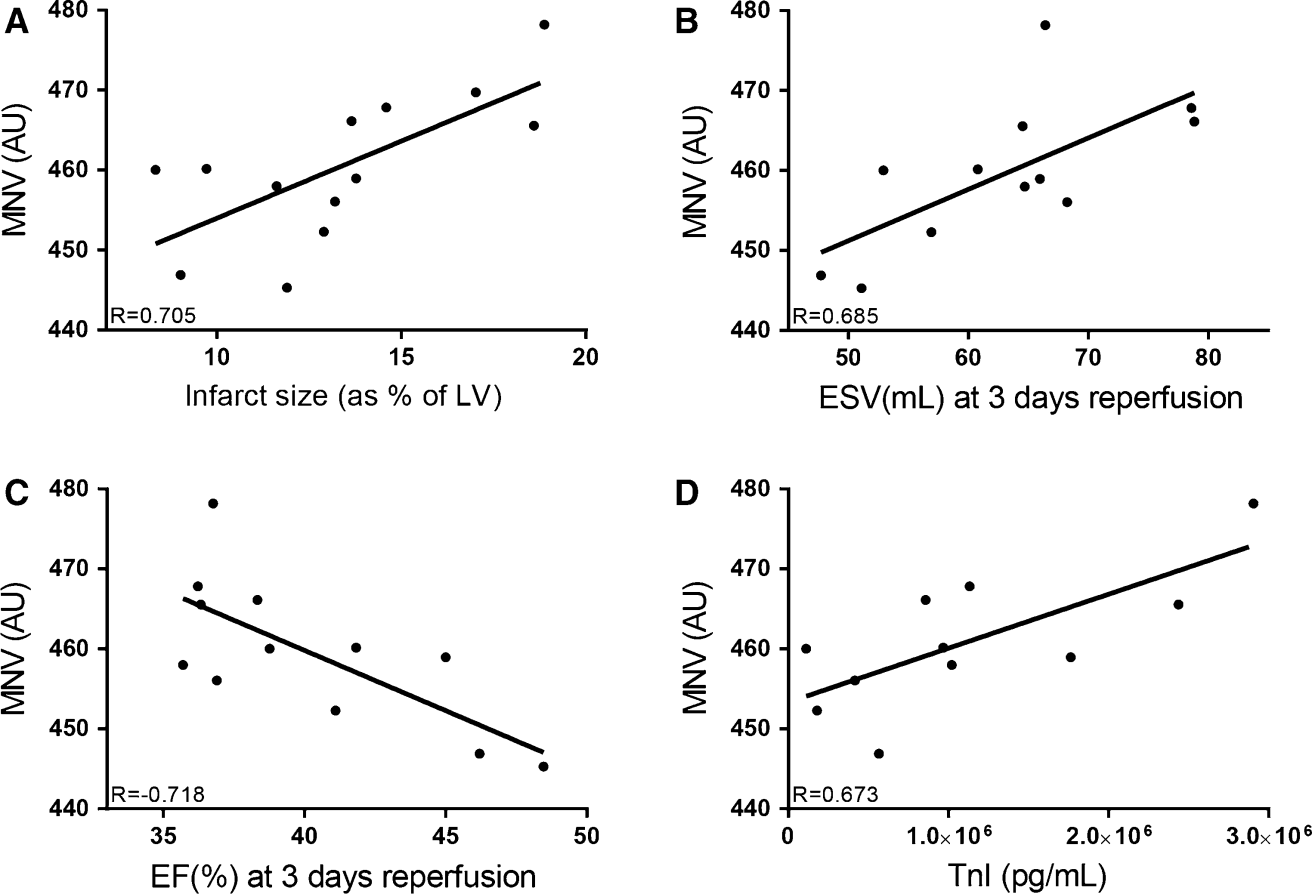
MNV correlates to cardiac damage in a porcine model of MI. **a** MNV correlates with infarct size as a percentage of the left ventricle. **b** MNV correlates with ESV measured by 3D-echocardiography 3 days after reperfusion. **c** MNV correlates with EF measured by 3D-echocardiography 3 days after reperfusion. **d** MNV correlates with TnI levels measured 2 h after reperfusion. *Pg* pictograms, *Ml* milliliter

To assess whether MNV was related to the amount of myocardial damage, MNV was correlated to infarct size as a percentage of the left ventricle (IS/LV) and cardiac function as assessed by 3D-echocardiography 72 h post-MI, combined with correlation to troponin I (TnI) levels measured in plasma 2 h post-MI. MNV measured from blood drawn at 15 min reperfusion correlated significantly with IS/LV (*R* = 0.705, *p* = 0.007) (Fig. 2a), end systolic volume (ESV) (*R* = 0.685, *p* = 0.014) (Fig. 2b) and inversely correlated with left ventricular ejection fraction (LVEF) (*R* = −0.718, *p* = 0.009) (Fig. 2c) measured by 3D-echocardiography on day 3. MNV also positively correlated with TnI (*R* = 0.673, *p* = 0.023) (Fig. 2d).

### Altered composition of circulating neutrophils contributes to MNV elevation

**Fig. 3:**
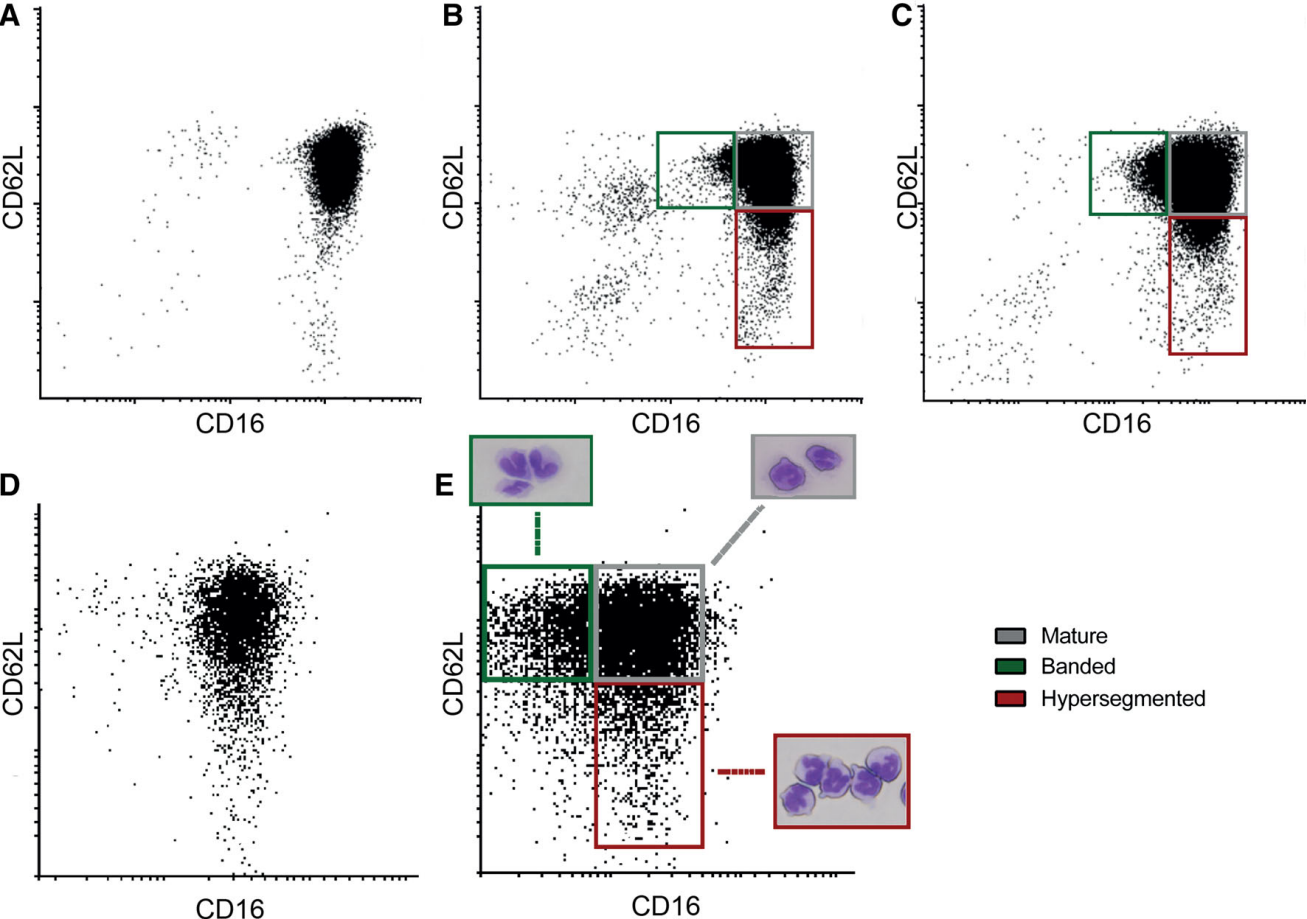
Scatterplots based on CD62L and CD16 expression of circulating neutrophils of STEMI patients and pigs subjected to MI. **a** Scatterplot of circulating neutrophils of a healthy control. **b**, **c** Scatterplot of circulating neutrophils of patients within 72 h post-MI. **d** Scatterplot of circulating neutrophils of a pig before subjection to MI. **e** Scatterplot of a pig 4 h after reperfusion with representative pictures of morphological differences between banded neutrophils (*green box*), mature neutrophils (*gray box*) and hypersegmented neutrophils (*red box*)

**Fig. 4:**
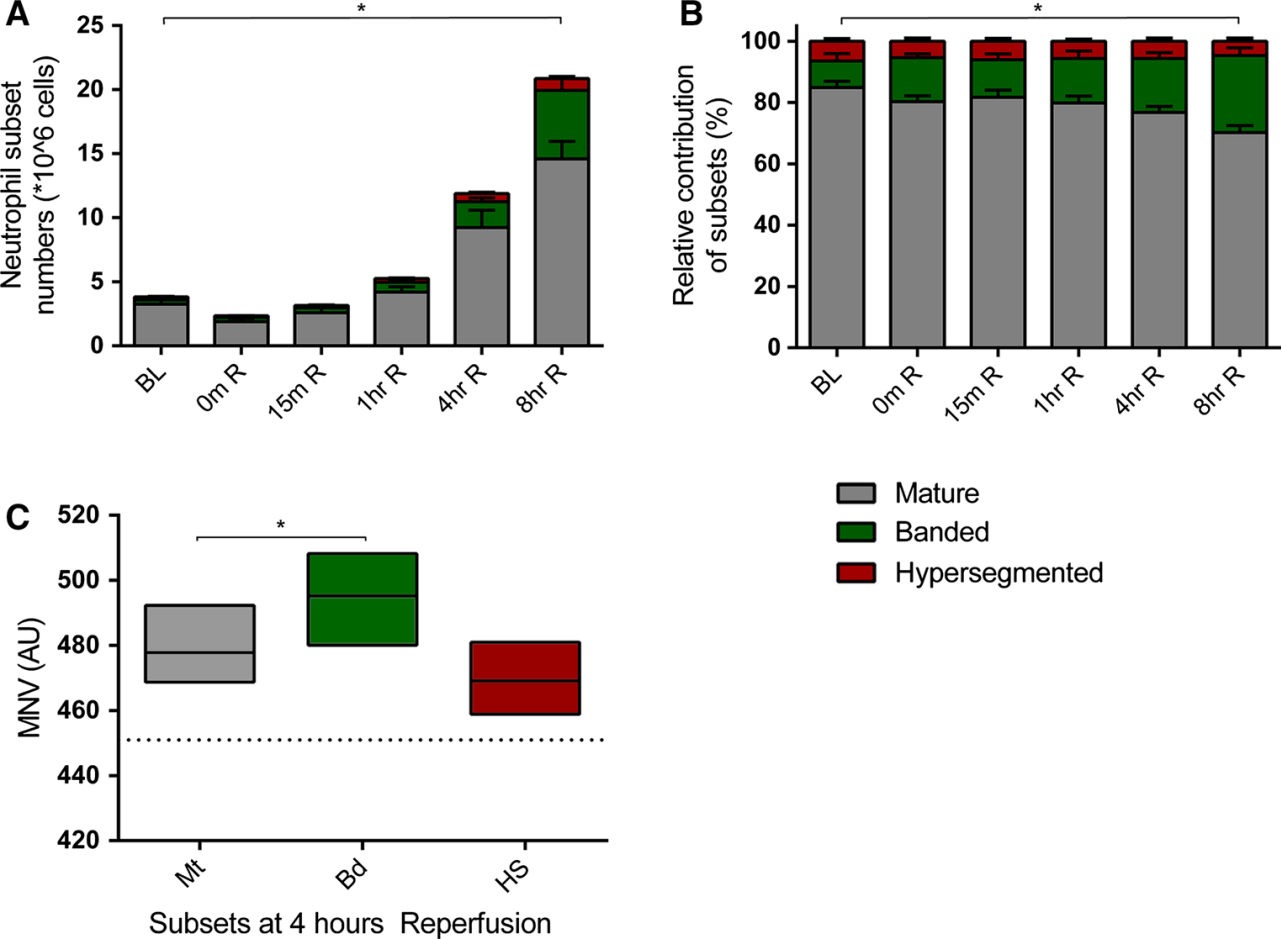
Banded neutrophils (relatively) increase during reperfusion and have a higher MNV in pigs subjected to MI. **a** Absolute increase in banded (*green*), mature (*gray*) and hypersegmented (*red*) neutrophils during reperfusion. **b** Relative increase of banded neutrophils (*green*) and decrease of mature neutrophils (*gray*) during reperfusion. **c** Banded neutrophils (*green*) have a higher MNV compared to mature neutrophils (*gray*), *dotted line* represents average MNV at baseline. *R* reperfusion, *Mt* mature, *Bd* banded, *HS* hypersegmented neutrophils, *p < 0.05

To investigate whether the increased MNV was due to the appearance of circulating neutrophil subsets, in particular banded and hypersegmented neutrophils, we performed CD62L/CD16 flow cytometry analysis of circulating neutrophils of a healthy control (Fig. 3a) and two STEMI patients (Fig. 3b, c). Since both patients revealed the presence of a more heterogeneous population, we also determined the presence of neutrophils subsets at baseline (Fig. 3d) and post-MI (Fig. 3e) in pigs. Consecutive sorting and visualization of the different subtypes confirmed the presence of banded (CD16^dim^/CD62L^high^), mature (CD16^high^/CD62L^high^) and hypersegmented (CD16^high^/CD62L^dim^) neutrophils in pigs post-MI (Fig. 3e). Quantification of subset numbers revealed an absolute increase in mature neutrophils (3.3 SD0.93 × 10^6^cells/mL vs. 15.3 SD2.97 × 10^6^cell/mL, *p* < 0.0001), banded neutrophils (0.36 SD0.16 × 10^6^cell/mL vs. 5.6 SD2.36 × 10^6^cells/ml, *p* = 0.001) and hypersegmented neutrophils (0.22 SD0.08 × 10^6^cells/ml vs. 0.82 SD0.39cells/ml, *p* = 0.011) between baseline and 8 h reperfusion in pigs subjected to MI (Fig. 4a). Moreover, relative contribution of subsets also changed over time. At 8 h reperfusion, mature neutrophils contributed less to the total population of circulating neutrophils than at baseline (70.8 SD5.6 % vs. 84.4 SD5.5 %, *p* = 0.008). On the other hand, the contribution of banded neutrophils increased when 8 h reperfusion was compared to baseline (25.2 SD6.4 % vs. 9.78 SD5.6 %, *p* = 0.006), while the contribution of hypersegmented neutrophils remained unchanged (3.93 SD1.91 % vs. 5.78 SD1.56 %, *p* = 0.152) (Fig. 4b). To assess whether this altered composition of circulating neutrophils could, in part, be responsible for an altered MNV, the MNV of different neutrophil subtypes was determined by an automated hematological analyzer. Post-MI, MNV of banded neutrophils was significantly higher than MNV of mature neutrophils (495 SD14 AU vs. 478 SD11 AU, *p* = 0.012) (Fig. 4c).

### Activation of neutrophils results in higher mean neutrophil volume

**Fig. 5:**
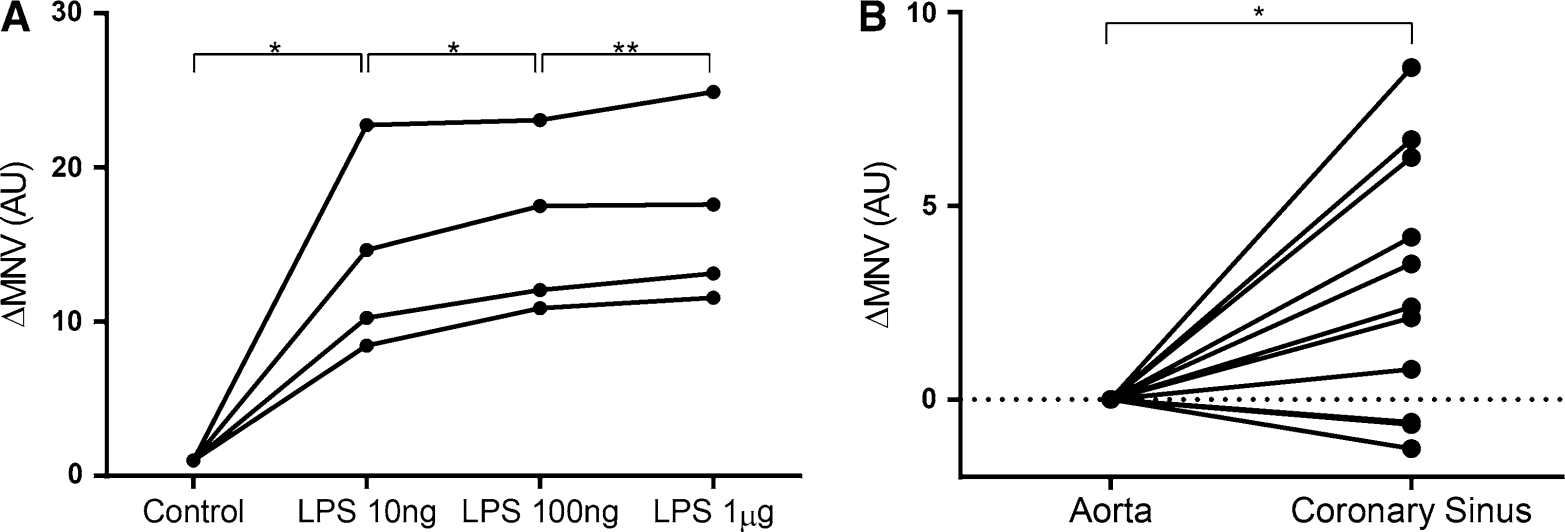
Local activation of neutrophils results in an increased MNV. **a** In vitro stimulation with LPS dose dependently increases MNV. **b** MNV in blood drawn from the coronary sinus is higher than MNV from simultaneously drawn blood from the aorta *p < 0.05, **p = 0.09

Not only banded neutrophils, but also mature neutrophils after MI showed a higher MNV than mature neutrophils at baseline, indicating the presence of another mechanism underlying these changes. Therefore, we assessed if local activation of neutrophils could alter MNV by stimulating whole blood of healthy pigs with 3 different doses of LPS (10, 100 ng, 1 µg) or PBS in vitro. After incubation of 2 h at 37 °C, a dose-dependent increase of MNV was seen (454 SD6.8 AU (control) vs. 470 SD8.7 AU (LPS 1 µg), *p* = 0.013) (Fig. 5a). Moreover, we determined if MNV was also altered due to the interaction between neutrophils and the damaged myocardium in vivo. To this extent, coronary sinus sampling was performed simultaneously with aortic sampling 15 min after reperfusion. MNV of neutrophils measured from coronary sinus blood was significantly higher than MNV of neutrophils from simultaneously collected arterial blood (463 SD7.6 AU vs 461 SD8.6 AU, *p* = 0.013) (Fig. 5b).

## Discussion

The detrimental effects of the inflammatory response post-MI are believed to outweigh the positive effects [44]. Indeed, multiple clinical studies have shown that different parameters of inflammation correlate with prognosis and adverse events after MI [19, 27, 33, 46]. Since neutrophils play a prominent role in myocardial ischemia–reperfusion injury [50], and MNV is known to be elevated in inflammation-related diseases [9, 29], we hypothesized that MNV could serve as a possible marker in the setting of MI. In this perspective, the current study sought to investigate whether MNV is elevated post-MI and what the possible explanation behind this elevation could be.

To the best of our knowledge, this is the first study to show that MNV is elevated in STEMI patients in response to myocardial infarction. Moreover, MNV correlates with CK levels, suggesting possible reflection of the amount of cardiac damage post-MI. Similar to the patient setting, MNV is elevated in pigs and MNV levels early after MI correlate with multiple parameters of cardiac damage measured after 3 days follow-up. These findings propose that MNV could withhold prognostic value in the setting of MI.

Additionally, we investigated for the first time, whether a change in MNV was the result of an altered neutrophil composition, or the activation of circulating neutrophils. It has recently been corroborated that migration of specific neutrophil subsets into the systemic circulation occurs in inflammation-related diseases [16, 40, 49, 51]. However, ours is the first study that identifies this phenomenon after MI in patients and morphologically confirmed these subsets in a porcine model of myocardial infarction.

Although previously suggested by others [54], we here provide the first direct evidence that this altered circulating neutrophil composition contributes to the elevated MNV. We have shown that banded neutrophils have a significantly higher MNV than mature neutrophils and account for more than a quarter of the circulating neutrophils post-reperfusion in pigs. Similar levels of banded neutrophils have been found in human subjects after in vivo LPS infusion, suggesting that our porcine model is representative for the human situation as well [40].

In contrast to the previous assumption that the increased MNV in inflammation-related diseases is only due to transmigration of banded neutrophils [10, 34, 54], we show that an altered subset composition is not the sole mechanism, since both in vitro and in vivo neutrophil activation results in significant MNV elevation. This phenomenon underscores that morphological characteristics of neutrophils could withhold great prognostic value post-MI, being directly and imminently influenced by the damaged myocardium.

To what extent both processes are exactly responsible for the change in MNV is hard to determine since they are very dynamic and time-dependent [52]. From our data and those of others, however, it is clear that the migration of subsets towards the systemic circulation occurs within a few hours from the initial event [40]. We also show that activation of neutrophils by the damaged myocardium already occurs during ischemia and early reperfusion. The contribution of banded neutrophils to the total neutrophil population is limited within this timeframe. This suggests that both processes overlap but that activation is probably playing a more dominant role in the first phase after cardiac ischemia. This seems logical since many different danger molecules are released from the damaged myocardium into the systemic circulation from the onset of ischemia onwards [30, 48, 50].

The current study advocates an association between MNV and the amount of damage inflicted after MI and may lead to further insight into the mechanism behind reperfusion damage and myocardial remodeling following ischemia. However, the true prognostic value of MNV could not be tested in the current study and needs to be addressed in future studies. Concomitantly, the correlation shown in our study between CK levels and MNV was not very strong. One of the main reasons is that we were limited to one single measurement in these patients, precluding a comparison between peak levels. Also, a considerable overlap between MNV of ischemic and non-ischemic patients may be appreciated. This renders MNV unlikely to become a diagnostic marker for acute coronary syndromes. Despite the limited value for diagnostic purposes, MNV could nevertheless provide prognostic value.

Since neutrophils are prominent players in myocardial reperfusion injury [50], it remains to be elucidated whether MNV is influenced by cardioprotective strategies, or could even be causally involved in the process of infarct expansion. A possible mechanism could be by enhancing the no-reflow phenomenon. As previously shown, neutrophil plugging is one of the most important causes of no-reflow after myocardial infarction [7]. Our study shows that neutrophils that are stimulated by the damaged myocardium gradually increase in size. Parallel to this observation, neutrophil activation also leads to microvascular obstruction. Whether larger neutrophils are generally more prone to congregate and cause no-reflow is unknown, but could be attributed to the above described events.

In our study, we established correlations with cardiac damage early after reperfusion. Additionally, the association of MNV with adverse events after MI may not only depend on cardiac damage, but also on comorbidity and risk factors not present in the large animal model we used. Apart from morphological differences of distinctive neutrophil subsets, also phenotypical differences have been identified [40, 42]. The distinctive role of the different neutrophil subsets in the setting of myocardial injury remains to be elucidated. Whether the presence of a heterogeneous population of neutrophils is associated with future adverse events after MI will be part of future studies and falls beyond the scope of our study.

In conclusion, the current study shows that MNV is elevated after MI and provides evidence that MNV predicts cardiac damage post-MI. Moreover, we show that MNV not only increases due to altered neutrophil composition, but also systemic neutrophil activation. Given the fast and inexpensive nature of MNV measurements with already clinically implemented devices, MNV may be an interesting parameter for future prognostic assessment in MI.
